# Social identity and capital income: A social psychological approach to identity economics using UK household data

**DOI:** 10.1111/bjso.70025

**Published:** 2025-12-03

**Authors:** Robin Bachmann, Ilka H. Gleibs, Liam Delaney

**Affiliations:** ^1^ Freie Universität Berlin Berlin Germany; ^2^ London School of Economics and Political Science London UK

**Keywords:** capital income, economic inequality, household data, identity economics, social identity, United Kingdom

## Abstract

Social identity research has yet to fully engage with identity economics. This article bridges the two by examining capital market participation and capital income inequality – a critical economic behaviour and a societal issue that remain understudied in social psychology. We integrate psychological concepts and metrics of social identity with large‐scale, representative UK data on household economics, encompassing 60,156 individuals and 130,598 observations from 2010 to 2023. Examining gender, ethnicity, education, occupation, politics, age and family as aspects of individuals' self‐concept, our findings show that between‐ and within‐person variations in these identities, beyond mere group memberships, were uniquely associated with both the presence and amount of capital income. Rather than reinforcing group membership effects, which could suggest adherence to group norms around capital market participation, our results highlight the importance of identity domains. Gender and ethnic identity were associated with lower capital income, whereas educational and political identity were linked to higher capital income. These patterns persisted across different groups and income strata. Importantly, the predictive power of social identities was comparable to traditional sociodemographic variables. This study extends social identity research to understudied economic behaviours and contributes to the emerging fields of identity economics and the psychology of inequality.

Social psychology has shown that social identities, our sense of self based on group memberships (Tajfel & Turner, 1979; Turner et al., 1987), profoundly influence many areas of life, yet economic consequences remain underexplored (Brown, 2020). Identity economics (Akerlof & Kranton, 2000, 2010) shows that people make economic sacrifices to preserve identity and belonging, which is especially relevant to the emerging field of the social psychology of inequality (Jetten & Peters, 2019; Kraus & Stephens, 2012; Manstead, 2018). Building on recent efforts to connect psychology and identity economics, we examine a novel case: capital market participation, a key driver of economic inequality (Piketty, 2014). Using economic household data and social identity measures, we examine how social identity might shape capital market behaviour and contribute to inequality.

## The social identity approach and identity economics

The *Social Identity Approach* (Tajfel & Turner, 1979; Turner et al., 1987) has been central in social psychology for understanding how people define themselves based on group memberships and behave in social contexts. Over decades, research has shown that social identities profoundly influence many aspects of life (Brown, 2020), including political (Mols et al., 2023; Reicher & Hopkins, 2000), organisational (Ashforth & Mael, 1989; Haslam, 2001) and health contexts (Haslam et al., 2018), and occasionally broader life outcomes (Gleibs et al., 2013; La Rue et al., 2024). However, the economic and financial implications of the social identity approach remain underexplored (Brown, 2020). Early evidence comes from the minimal group experiment itself (Tajfel et al., 1971), in which participants favoured their own group when allocating tokens. While originally designed to study intergroup behaviour, this paradigm can also be seen as a form of economic decision‐making. Later studies using real monetary stakes have replicated and extended these findings, primarily in economics rather than psychology (Chen & Li, 2009).

Traditional economics, focused on individual utility maximisation, has been challenged by behavioural economics (Kahneman & Tversky, 1979; Thaler, 2016), drawing extensively on psychological research (Camerer, 1999). However, behavioural economics – especially behavioural finance (Barberis & Thaler, 2003) – has been criticised for neglecting social context, prompting calls for ‘social finance’ (Hirshleifer, 2015), investigating social psychological factors in economic behaviour. Akerlof and Kranton's *Identity Economics* (2000, 2010) advances this by linking economic decisions to social identities. They argue that incorporating identity into economic models can (1) explain ‘maladaptive or even self‐destructive’ behaviour, (2) introduce ‘a new type of externality’ and (3) reveal ‘a new way preferences can be changed’ (Akerlof & Kranton, 2000, p. 717). For instance, identity can explain why individuals in poverty may spend on luxury goods despite financial constraints (Walasek & Brown, 2015), why men may oppose women's financial independence even when it benefits the household (Bucher‐Koenen & Lusardi, 2011), or why some people pursue entrepreneurial ventures (Murnieks et al., 2014). Drawing on these examples, Akerlof and Kranton (2000) argue that, given its economic consequences, identity may be ‘the most important ‘economic’ decision people make’ (p. 717).

In economics, identity economics has been applied to education, work, market behaviour (Charness & Chen, 2020; Shayo, 2020) and financial decision‐making at private (Benjamin et al., 2010; Morris et al., 2008), corporate (Fisman et al., 2017) and governmental levels (Guo et al., 2021). Though rooted in social psychology, it has received little attention there, with a recent exception from identity leadership research (Steffens et al., 2020). Yet, identity economics may be particularly relevant to the growing field of social psychology of inequality (Jetten & Peters, 2019; Kraus & Stephens, 2012; Manstead, 2018), which explores the intersection of social psychology and economic contexts. Inequality can amplify intergroup biases also in financial decisions (Connor et al., 2020; Peters & Jetten, 2023), while adverse conditions (Haushofer & Fehr, 2014) and internalised socioeconomic status (Sheehy‐Skeffington, 2020) can impair decision‐making, perpetuating poverty. Although much is known about how inequality affects identity, group dynamics and poverty‐linked financial behaviour, less is understood about how social identity shapes wealth‐building decisions, such as capital market participation.

## An identity perspective to capital market participation and capital income inequality

Our research examines capital market participation, the holding of stocks, bonds and other investment products. This behaviour is central to understanding economic inequality, as capital ownership is a major driver of widening wealth gaps (Piketty, 2014). However, it has received little attention in psychology because it is concentrated among less‐studied higher‐income groups (Leckelt et al., 2019). Moreover, social psychology tends to frame inequality in terms of disadvantages rather than advantages, which can obscure how privileges sustain disparities (Bruckmüller et al., 2017; Phillips et al., 2022). Capital market participation thus exemplifies an overlooked privileged economic behaviour.

Recent UK data (UK Office of National Statistics, 2023) highlights stark capital income disparities, affecting not only lower but also middle classes. While 90% of households earn just 1%–3% of income from capital, the top 10% earn 8%. In absolute terms, the poorest decile makes £200 annually from investments, the richest £16,500 ‐ over twice the total income of the poorest. Financial behaviour, particularly capital market participation, contributes to this gap. Many European households, despite having the financial means, either do not invest in capital markets or allocate far less to them than to bank deposits (European Fund and Asset Management Association [EFAMA], 2024). Globally, even though stock markets exist in over 95% of countries, the average participation rate across 70 studied nations is just 8% (Grout et al., 2009), underscoring the widespread nature of this challenge.

Economic research typically explains non‐participation in capital markets through factors such as participation costs – money, time, effort, or required knowledge (Guiso et al., 2003). Yet, generally low participation rates remain puzzling for standard economic models, since foregoing capital returns runs counter to the principle of utility maximisation (Haliassos, 2003). Behavioural economics addresses part of this gap by highlighting psychological determinants such as risk and time preferences (Donkers & Van Soest, 1999; Van der Heijden et al., 2012), locus of control (Salamanca et al., 2020) and trust and social capital (Balloch et al., 2015). An identity perspective adds a social dimension, answering calls for more research on ‘social finance’ (Hirshleifer, 2015).

Economic research highlighted group disparities in capital income. In the UK, participation is lower among less‐educated, lower‐occupation, younger, female and ethnic‐minority individuals (UK Department for Work and Pensions, 2022) and gaps persist even after controlling for general income (Jianakoplos & Bernasek, 1998; Painter et al., 2016). They partly reflect psychological and cultural factors. For example, women exhibit lower risk tolerance and financial confidence (Bucher‐Koenen et al., 2021; Jianakoplos & Bernasek, 1998; see also Barber & Odean, 2001), and East German women – shaped by more gender‐equal roles in the former GDR – take greater financial risk than West German women (Struewing & Jirjahn, 2019). Yet, these factors do not fully explain the impact of groups.

Singular studies have also examined how the perception of groups itself shapes investment behaviour. Henkel and Zimpelmann (2023) found that changing how investors are seen can change people's participation in capital markets. Morris et al. (2008) showed that conservative political identity salience led to conservative investments. Benjamin et al. (2010) found that identity salience influenced time and risk preferences, but only for race not for gender identity. While these studies demonstrate social identity's causal effects on investment behaviour, only the first directly addresses capital market participation, and none assess how typical social identities influence participation at the broader societal level.

## The present study

Bridging the social identity approach and identity economics, our study is the first to estimate the societal impact of social identity on capital market participation. We use large‐scale, representative household data from *Understanding Society: The UK Household Longitudinal Study* (UKHLS; University of Essex, Institute for Social and Economic Research, 2024). Across five waves (2010–2023), in addition to the typical sociodemographic information, the UKHLS asked respondents how important gender, ethnicity, education, occupation, political beliefs, age and family were for their self‐concept. This makes it a unique dataset for investigating social identity in a psychological sense and for advancing this perspective in the study of capital market behaviour.

Identity economics has so far largely equated social identity with group membership – whether someone belongs to a social group – (Akerlof & Kranton, 2000, 2010), while household studies of capital market participation have relied solely on sociodemographic variables framed as identity markers (Barber & Odean, 2001; Bucher‐Koenen et al., 2021; Jianakoplos & Bernasek, 1998; Painter et al., 2016). Psychological research, however, defines social identity not merely as group membership but as the personal ‘significance attached to that membership’ (Tajfel, 1981, p. 255). This distinction is important because analyses based solely on sociodemographics may capture disparities driven by external factors – for example, financial institutions discriminating against lower‐income groups. When linked to social identity, however, such differences more directly reflect personal financial decision‐making and behavior linked to the importance of the group membership. While persistent group‐related disparities in capital income are well‐documented, it remains unclear how social identities, in their psychological sense, relate to capital income. Thus, our study asks:

*RQ1*: How are social identities, in a psychological sense, associated with capital income?

By distinguishing between group membership and its personal significance, we make a substantial contribution to an identity perspective on capital market participation. Our study goes further by differentiating between *identity categories* (Turner et al., 1987) – specific self‐aspects that divide people into groups (e.g., men, women, others) – and what we term *identity domains* – broader self‐aspects that span across groups (e.g., gender). An individual may primarily think of themselves in terms of a specific category (e.g., ‘I am a man.’), but for research, it is important to separate effects tied to specific categories (identifying as man, woman, other differently) from effects tied to the domain itself (the importance of gender for an individual's identity). This distinction also reflects two potential pathways how social identity might influencing behaviour: through norms associated with social categories or through priorities linked to identity domains. We elaborate on these pathways in the following.

A classic psychological mechanism linking identity to behaviour is adherence to group norms. Individuals tend to adjust their behaviour to align with the expectations of others – particularly those with whom they strongly identify (Terry et al., 1999). Identity economics also frames norms as the behavioural side of identities (Akerlof & Kranton, 2000, 2010; Charness & Chen, 2020) and sees adherence to group norms – beside ingroup bias – as central mechanism, especially in private economic decisions (Shayo, 2020). This perspective could help explain well‐documented disparities in capital market participation. In the UK, it is lower among women, ethnic minorities, individuals with lower levels of education or occupational status, those with left‐leaning political affiliations, younger people, and those without a legal partnership (UK Department for Work and Pensions, 2022). Members of these groups may participate less because they conform to non‐participation norms, arising either from low prevalence of participation (a descriptive norm; Cialdini et al., 1990) or negative perceptions of investments (Henkel & Zimpelmann, 2023). Building on these patterns, we investigate whether identification amplifies group disparities in capital income. Specifically, we ask:

*RQ2*: Is identification with more‐participating groups (men, ethnic majority, high education and occupation level, and politically right‐leaning, older age and partnered) associated with higher capital income, while identification with less‐participating groups (women, ethnic minority, low education and occupation level, and politically left‐leaning, younger age and unpartnered) associated with lower capital income?

A second pathway through which social identity may shape participation is the importance of identity domains. According to the *Multiple Self‐Aspect Framework* (MSAF; McConnell, 2011), individuals' identities consist of clusters around multiple aspects, each varying in salience and significance across social contexts. Linking this framework to socioeconomic backgrounds, Easterbrook et al. (2020) found that higher socioeconomic status was associated with greater importance of socioeconomic identities (e.g., education, occupation), while lower socioeconomic status correlated with sociocultural identities (e.g., gender, ethnicity). This aligns with social identity theory, suggesting that individuals downplay lower‐status characteristics such as lower socioeconomic status (Tajfel & Turner, 1979), and with identity economics, which suggests that disadvantaged individuals derive more identity utility from sociocultural identities (Akerlof & Kranton, 2000, 2010). Together, these findings suggest that the importance of particular identity domains may systematically influence priorities and decision‐making.

We extend this reasoning to economic behaviour, proposing that the importance of identity domains influences capital market participation. Individuals prioritising socioeconomic identity may adopt goals and behaviours aligned with that domain, such as financial and investment activity. Conversely, those prioritising sociocultural identities may derive satisfaction from them in ways that substitute for or compete with financial activities, reducing capital market engagement. Unlike norm adherence, this mechanism can operate across groups with differing capital market participation norms. While prior capital market participation studies have not tested this directly, there is some preliminary evidence for this mechanism: While Changwony et al. (2015) found that in the UK, Conservative and Liberal affiliation was associated with capital market participation, Bonaparte and Kumar (2013) showed that in the US, any party affiliation – regardless of ideology – was associated with participation. These findings imply that an identity domain can shape financial behaviour independently of specific social categories and associated norms. More broadly, we ask:

*RQ3*: Is the importance of socioeconomic identity domains (education, occupation, political beliefs) associated with higher capital income, while that of sociocultural identity domains (gender, ethnicity) associated with lower capital income, regardless of the specific group memberships within these identity domains?

Finally, we explore whether the influence of social identity varies by income level. Prior research suggests that while gender and ethnic group membership shape capital market participation, their impact weakens as income rises (Jianakoplos & Bernasek, 1998; Painter et al., 2016). We explored whether this also applies to social identities, as higher‐income individuals may experience weaker group norms discouraging participation due to higher overall participation rates, or may have additional identity domains – such as education and occupation – from which they can derive identity gains, reducing the influence of other identity domains – such as gender and ethnicity. Given limited research on this topic, this analysis remains exploratory. We ask:

*RQ4*: Is the association of social identities with capital income moderated by total individual income levels?

## METHOD

We used large‐scale representative household data from *Understanding Society: The UK Household Longitudinal Study* (*UKHLS*; University of Essex, Institute for Social and Economic Research, 2024), which included social identity questions in five waves: *Wave 2* (2010–11), *Wave 5* (2013–14), *Wave 8* (2016–17), *Wave 11* (2019–20) and *Wave 14* (2022–23).

### Measures

*Social identities* were measured with seven items (‘How important are various things to your sense of who you are: gender, ethnic background, education, profession, political beliefs, age/ life stage, family?’) rated on a four‐point Likert scale ranging from 1 (*not at all important to my sense of who I am*) to 4 (*very important to my sense of who I am*).

*Group memberships* were measured using sociodemographic variables with an interpretable set of factor levels: *gender* (male, female), *ethnicity* (White, Black, Asian, other), *education level* (basic, high school, university),^1^ *occupation level* (elementary, administrative, skilled, professionals),^2^ *occupation status* (employed, self‐employed, unemployed, not‐working, retired), *political affiliation* (none, Conservative, Labour, Liberal, Green, other),^3^ *age* (<25, 25–39, 40–54, 55–69, >70), *civil status* (single, partnered, separated) and *children* (none, one/two, three and more).^4^

*Capital income* was measured as *monthly individual income from savings and investments*. A binary variable indicated whether there was any such income and a continuous variable indicated the amount of income. Furthermore, *total income* was measured as *total monthly individual net income*, encompassing also income from labour, pension, social and private benefits. Continuous income measures were logarithmised for regressions.

### Sample

For each wave, we included all respondents who completed the full set of identity questions. Participants with missing income information were excluded (<1% of the sample). No additional exclusions were applied. Missing values in group membership variables were coded as a separate factor level. Because the analysis focused on individual capital income, we included all household members aged 16 and older, rather than restricting the sample to the household head who typically reports household finances. The sample was unbalanced: respondents were included even if they did not participate in all five waves. The final sample consisted of *N*_Ind_ = 60,156 individuals and *N*_Obs_ = 130,598 observations.

### Analysis

We analysed research questions using mixed‐effects linear regression models (McNeish & Kelley, 2019). The longitudinal structure of the data allowed us to distinguish between‐person associations (whether individuals with higher average identity scores across waves also had higher average capital income) from within‐person associations (whether deviations from an individual's own average identity score were related to deviations from their average capital income). Within‐person associations are generally less confounded by stable individual characteristics, as each person serves as their own control.

Specifically, the presence of any capital income and the amount of capital income (DVs) was regressed on between‐ and within‐person components of the seven identity scores (IVs):

$${\mathrm{DV}}_{\mathrm{it}}=\beta_{0}+\beta_{\text{1within}}\;\left({\mathrm{IV}}_{1\mathrm{it}}-\text{mean}\left({\mathrm{IV}}_{1i}\right)\right)+\beta_{\text{1between}}\;\text{mean}\left({\mathrm{IV}}_{1i}\right)+\ldots +u_{i}+\varepsilon_{\mathrm{it}}$$

where *u*_*i*_ is the random intercept for participant *i*, and *ε*_*it*_ is the residual error. All identity terms were included simultaneously. Both DVs and IVs were standardised, with IVs scaled after decomposition. Models were estimated using Restricted Maximum Likelihood (REML). The full unbalanced sample was used – only participants with at least two observations contributed to within‐person estimates. The linear model was applied to both the amount of capital income (continuous) and the presence of any capital income (binary). Although psychologists might expect logit or probit models for binary outcomes, linear models have been shown to yield more robust estimates and are more commonly used for binary variables in economic research (Gomila, 2021).

We assessed associations between social identities and capital income (RQ1) first without additional controls (Model 1.1), then controlled for total income at both the between‐person and within‐person levels (Model 1.2), and finally added all group membership variables at the between‐person level (Model 1.3). For all subsequent analyses, total income and group memberships were retained as controls.^5^

To investigate whether identification with groups characterised by different participation norms was associated with capital income (RQ2), we included interactions between identity components (between‐ and within‐person) and the respective group variables (Model 2). Group variables were treated as categorical factors, with one category serving as the reference group. Evidence for norm effects was evaluated based on the direction of interactions and of associations within groups. For instance, if a gender identity was more strongly associated with lower capital income for women than men, this was considered weak evidence of a norm effect, while if it was associated with lower capital income for women but higher capital income for men, this was interpreted as strong evidence of a norm effect.

To assess whether specific identity domains were associated with capital income (RQ3), we reexamined both Model 1.3 and Model 2. Evidence for domain effects was evaluated based on associations across and within groups. For example, a general association of gender identity with lower capital income constituted weak evidence of a domain effect, while lower capital income for both men and women represented strong evidence.

Notably, strong versions of norm and domain effects are mutually exclusive, whereas weak versions can co‐occur. For example, if gender identity is associated with lower capital income for both men and women (strong domain effect), it cannot simultaneously be associated with lower capital income for women and higher capital income for men (strong norm effect). However, in the first case, gender identity can still be more strongly associated with lower income for women than men (weak norm effect), while in the second case, it can still have a general association with lower capital income across genders (weak domain effect).

Finally, we tested whether the association of identities with capital income varied across income levels (RQ4) by including interactions between between‐person total income and both between‐ and within‐person identity components (Model 3).

## RESULTS

On average, respondents rated all identities as *fairly important* (*M* = 2.9–3.0, *SD* = 0.9–1.0), with ethnicity (*M* = 2.4, *SD* = 1.0) and politics (*M* = 2.2, *SD* = 1.0) rated lower and family higher (*M* = 3.6, SD = 0.7), reflecting the overall relevance of identity in the population. Correlations between identities were weak to moderate (*r* = .10–.41), with stronger correlations between educational and occupational identity (*r* = .54) and gender and ethnic identity (*r* = .51), and gender and age identity (*r* = .61). These patterns align with previous research, which has combined socioeconomic and sociocultural dimensions (Easterbrook et al., 2020); our analysis, however, examined each identity separately.

While capital income participation was relatively high (33% of observations), the average amount was relatively low (*M* = £108, *SD* = £500 per month), indicating that our measures captured even small amounts and supporting the distinction between presence and amounts (*r* = .17). Capital income was, as one might expect, positively but only moderately correlated with total net income (*r* = .32). Table 1 reports all descriptive statistics and correlations.

**TABLE 1:** Descriptive statistics and correlations.

Variable	*M*	*SD*	1	2	3	4	5	6	7	8	9
*Identities*											
1. ID gender	2.9	1.0									
2. ID ethnicity	2.4	1.0	.51***								
3. ID education	2.9	0.9	.27***	.34***							
4. ID occupation	3.0	0.9	.23***	.27***	.54***						
5. ID politics	2.2	1.0	.28***	.40***	.29***	.21***					
6. ID age	3.0	0.9	.61***	.41***	.32***	.28***	.26***				
7. ID family	3.6	0.7	.31***	.24***	.20***	.20***	.10***	.29***			
*Income*											
8. Capital (any)^a^	0.33	0.47	−.05***	−.05***	.03***	.00	.03***	−.04***	−.04***		
9. Capital (£)^a^	36	291	−.01***	−.02***	.01*	.01*	.01***	−.01***	−.01**	.17***	
10. Total (£)^b^	1683	1.91	−.05***	−.04***	.03***	.07***	.03***	−.05***	−.01***	.15***	.32***

*Note*: *N*_Obs_ = 130,598.

Abbreviation: ID, identity.

^a^ Monthly individual income from savings and investments.

^b^ Total monthly individual net income.

*p < .05, **p < .01, ***p < .001.

### Associations between social identities and capital income

Table 2 presents the associations between social identities and capital income (RQ1), without controls (Model 1.1), with total income (Model 1.2), and additionally with group membership controls (Model 1.3). Between persons, gender, ethnic, occupational, age and family identity were associated with both lower likelihood and amounts of capital income, whereas educational and political identity were associated with both higher likelihood and amounts of capital income. Within persons, educational, occupational, political and age identity were associated with higher capital income (occupation only its likelihood), whereas gender, ethnic and age identity showed no association. Notably, while educational and political identity had the same positive association with capital income both between and within persons, occupational and family identity reversed from negative to positive associations.

**TABLE 2:** Capital income predicted by identities (Model 1).

Predictor	Capital income (any)			Capital income (amount)		
	Model 1.1	Model 1.2	Model 1.3	Model 1.1	Model 1.2	Model 1.3
	*β* (SE)	*β* (SE)	*β* (SE)	*β* (SE)	*β* (SE)	*β* (SE)
*Between effects*						
ID gender	−.022*** (0.005)	−.021*** (0.005)	−.017*** (0.004)	−.020*** (0.005)	−.018*** (0.005)	−.016*** (0.004)
ID ethnicity	−.063*** (0.004)	−.053*** (0.004)	−.013** (0.004)	−.054*** (0.004)	−.043*** (0.004)	−.018*** (0.004)
ID education	.054*** (0.004)	.067*** (0.004)	.029*** (0.004)	.051*** (0.004)	.064*** (0.004)	.030*** (0.004)
ID occupation	−.019*** (0.004)	−.030*** (0.004)	−.015*** (0.004)	−.018*** (0.004)	−.029*** (0.004)	−.006 (0.004)
ID politics	.061*** (0.004)	.059*** (0.004)	.014*** (0.004)	.068*** (0.004)	.065*** (0.004)	.015*** (0.004)
ID age	−.026*** (0.004)	−.015*** (0.004)	.007 (0.004)	−.037*** (0.005)	−.025*** (0.004)	.001 (0.004)
ID family	−.029*** (0.004)	−.040*** (0.003)	−.038*** (0.003)	−.021*** (0.004)	−.033*** (0.004)	−.031*** (0.003)
*Within effects*						
ID gender	−.003 (0.003)	−.003 (0.003)	−.003 (0.003)	−.002 (0.002)	−.002 (0.002)	−.002 (0.002)
ID ethnicity	−.004 (0.002)	−.004 (0.002)	−.004 (0.002)	−.001 (0.002)	−.001 (0.002)	−.001 (0.002)
ID education	.006* (0.002)	.007** (0.002)	.007** (0.002)	.006** (0.002)	.009*** (0.002)	.009*** (0.002)
ID occupation	.006* (0.002)	.006* (0.002)	.006* (0.002)	.002 (0.002)	.001 (0.002)	.001 (0.002)
ID politics	.007** (0.002)	.006* (0.002)	.006* (0.002)	.006** (0.002)	.005* (0.002)	.005* (0.002)
ID age	.000 (0.003)	.000 (0.003)	.000 (0.003)	−.002 (0.002)	−.002 (0.002)	−.002 (0.002)
ID family	.007** (0.002)	.007** (0.002)	.007** (0.002)	.005* (0.002)	.005* (0.002)	.005* (0.002)
*Controls*						
Total income^a^		Yes	Yes		Yes	Yes
Groups^b^			Yes			Yes
*Random effects*						
Var_Residual_	.62	.61	.61	.55	.54	.54
Var_Ind_	.36	.33	.26	.42	.39	.30
ICC	.37	.35	.30	.44	.42	.36
*R* ^2^_Marginal_	.011	.034	.121	.010	.040	.142
*R* ^2^_Conditional_	.375	.374	.382	.441	.442	.449

*Note*: *N*_Obs_ = 130,598, *N*_Ind_ = 60,156. Mixed effect regression models with random intercepts for individuals. Standardised estimates and standard errors.

Abbreviation: ID, identity.

^a^ Total monthly individual net income at between‐ and within‐level (logarithmised).

^b^ Group memberships: gender, ethnicity, education, occupation level and status, party affiliation, civil status and children.

*p < .05, **p < .01, ***p < .001.

Associations remained largely stable when introducing controls: adjusting for total income had little effect (Model 1.2), whereas adjusting for group membership (Model 1.3) reduced between‐person associations of ethnic, educational and political identity. Occupational and age identity became insignificant (occupational identity only for the amount). Between‐person associations were, as one might expect, stronger than within‐person associations. The ICCs indicated that 30%–44% of the variance in capital income was attributable to between‐person differences. Although the marginal *R* ^2^ for identity variables alone was small, at around 1%, it was notable given that a wide range of sociodemographic variables explained only 12%–14% of the variance.

Three key findings emerge. First, social identity was associated with both the presence and amount of capital income between and within persons.^6^ Second, associations were robust when controlling for total income and sociodemographic factors, indicating that they reflected capital income rather than general income, and psychological identification rather than mere group membership. Third, while identity variables explained a small share of the total variance, their contribution is substantial relative to the explanatory power of sociodemographic variables.

### Group norms vs. identity domains

**FIGURE 1:**
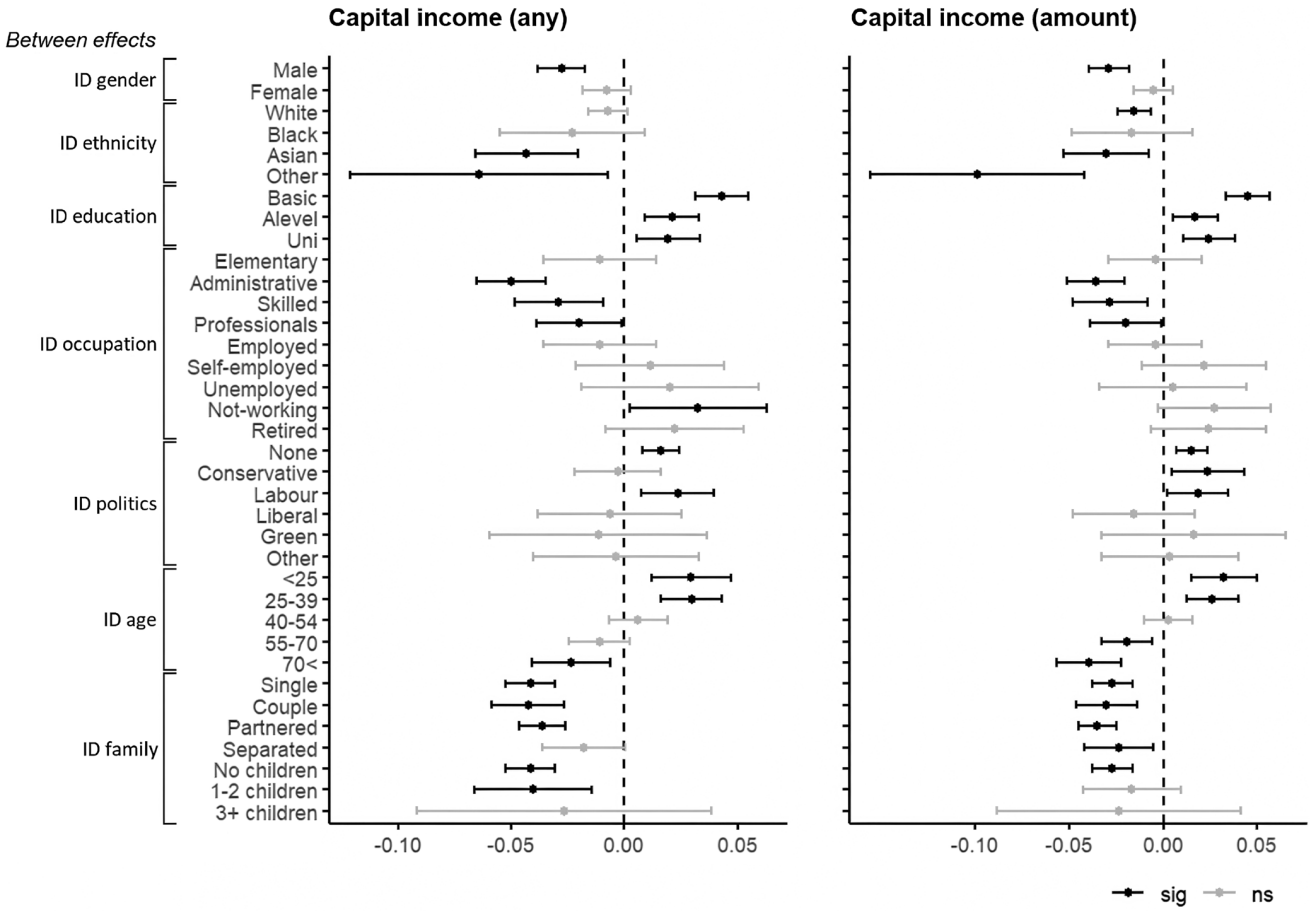
Capital income predicted by identities interacting with group membership (Model 2). Standardised estimates and confidence intervals of identity effects within groups based on mixed effect regression models with interactions between identity and group variables (Model 2). Models control for total net income at between‐ and within‐level. Shown are between effects only. See Table S2 for full statistics. ID, identity.

Figure 1 shows the associations between social identity and capital income across several sociodemographic groups, based on the regression model including the interaction between social identity and group membership variables (Model 2, see Table 3 for full statistics). This analysis examined two different pathways through which social identity might influence capital income: adherence to group norms (RQ2) and importance of identity domains (RQ3). Evidence for adherence to group norms was very limited. Between persons, individuals with Asian and other backgrounds (vs. White), having lower capital income, also had a stronger negative association of ethnic identity with capital income – though this was not the case for Black ethnic backgrounds. Self‐employed and retired individuals (vs. employed), having higher capital income, also had a less negative association of occupational identity with capital income. Within persons, individuals in skilled jobs and professions (vs. lower occupation levels), as well as partnered and couples (vs. singles), having higher capital income, had a positive association of occupational identity with capital income. However, in contrast to norm adherence, between persons, men, higher‐educated, higher‐ranking, right‐leaning, older and partnered individuals (vs. their counterparts), having more capital income, had a weaker positive – or even a stronger negative – association of gender, educational, occupational, political, age or family identity with capital income. Within persons, lower‐educated, lower‐ranking and left‐leaning individuals, having lower capital income, did not have a less positive association of education, occupation or political identity with capital income.

**TABLE 3:** Capital income predicted by identities and group memberships (Model 2).

Predictor	Capital income (any)			Capital income (amount)		
	Main	× ID_between_	× ID_within_	Main	× ID_between_	× ID_within_
	*β* (SE)	*β* (SE)	*β* (SE)	*β* (SE)	*β* (SE)	*β* (SE)
*Fixed effects*						
ID gender		−.028*** (0.005)	−.006^+^ (0.003)		−.029*** (0.005)	−.005 (0.003)
Female^a^	.007 (0.007)	.020** (0.006)	.006 (0.004)	−.046*** (0.007)	.024*** (0.006)	.005 (0.004)
ID ethnicity		−.007 (0.005)	−.004 (0.003)		−.015*** (0.005)	−.001 (0.002)
Black^b^	−.234*** (0.022)	−.016 (0.017)	.010 (0.012)	−.208*** (0.023)	−.001 (0.017)	−.003 (0.012)
Asian^b^	−.149*** (0.015)	−.036** (0.012)	−.003 (0.008)	−.087*** (0.015)	−.015 (0.012)	.005 (0.008)
Other^b^	−.135*** (0.034)	−.057^+^ (0.029)	−.028 (0.022)	−.063^+^ (0.034)	−.084** (0.029)	−.031 (0.021)
ID education		.043*** (0.006)	.010** (0.004)		.045*** (0.006)	.008* (0.004)
A‐level^c^	.131*** (0.008)	−.022** (0.008)	−.002 (0.005)	.136*** (0.008)	−.028*** (0.008)	.002 (0.005)
Uni^c^	.330*** (0.010)	−.024** (0.009)	−.008 (0.006)	.340*** (0.010)	−.021* (0.009)	−.001 (0.005)
ID occupation		−.011 (0.013)	.010 (0.010)		−.004 (0.013)	−.007 (0.009)
Admin.^d^	.083*** (0.016)	−.039** (0.014)	−.005 (0.011)	.062*** (0.016)	−.032* (0.014)	.004 (0.010)
Skilled^d^	.156*** (0.017)	−.018 (0.016)	.000 (0.012)	.099*** (0.017)	−.024 (0.016)	.016 (0.011)
Profes.^d^	.217*** (0.017)	−.009 (0.015)	.005 (0.012)	.175*** (0.017)	−.016 (0.016)	.012 (0.011)
Self‐empl.^e^	.059*** (0.012)	.022^+^ (0.012)	−.013 (0.008)	.141*** (0.012)	.026* (0.012)	−.006 (0.007)
Unempl.^e^	−.138*** (0.018)	.031^+^ (0.016)	−.021 (0.013)	−.026 (0.018)	.009 (0.016)	−.013 (0.012)
Not‐working^e^	−.080*** (0.012)	.043*** (0.010)	−.003 (0.008)	−.002 (0.012)	.031** (0.010)	.004 (0.008)
Retired^e^	.215*** (0.014)	.033** (0.010)	−.001 (0.007)	.326*** (0.015)	.028** (0.010)	.007 (0.006)
ID politics		.016*** (0.004)	.009** (0.003)		.015*** (0.004)	.006* (0.003)
Conservative^f^	.131*** (0.010)	−.019^+^ (0.010)	−.010^+^ (0.006)	.116*** (0.010)	.009 (0.010)	.002 (0.006)
Labour^f^	−.029** (0.010)	.008 (0.009)	−.004 (0.006)	−.085*** (0.010)	.003 (0.009)	−.005 (0.005)
Liberal^f^	.268*** (0.016)	−.022 (0.017)	−.004 (0.010)	.206*** (0.016)	−.031^+^ (0.017)	−.008 (0.009)
Green^f^	.170*** (0.024)	−.027 (0.025)	−.015 (0.015)	.082*** (0.024)	.001 (0.025)	.008 (0.014)
Other^f^	−.135*** (0.022)	−.020 (0.019)	−.025 (0.019)	−.092*** (0.022)	−.012 (0.019)	−.016 (0.018)
ID age		.029** (0.009)	−.006 (0.010)		.032*** (0.009)	−.009 (0.010)
25–40^g^	−.108*** (0.014)	.000 (0.011)	.009 (0.011)	−.097*** (0.014)	−.006 (0.011)	.002 (0.010)
40–55^g^	−.048** (0.015)	−.023* (0.010)	.003 (0.011)	−.009 (0.015)	−.030** (0.010)	.009 (0.010)
55–70^g^	.163*** (0.016)	−.040*** (0.011)	.006 (0.011)	.216*** (0.016)	−.052*** (0.011)	.004 (0.010)
70+^g^	.230*** (0.021)	−.053*** (0.012)	.011 (0.012)	.277*** (0.021)	−.072*** (0.012)	.016 (0.011)
ID family		−.042*** (0.006)	.005 (0.005)		−.027*** (0.006)	.007 (0.005)
Couple^h^	−.013 (0.012)	−.001 (0.010)	.006 (0.008)	−.021^+^ (0.013)	−.003 (0.010)	.002 (0.007)
Partnered^h^	.056*** (0.010)	.005 (0.007)	.006 (0.006)	.053*** (0.010)	−.008 (0.007)	.000 (0.005)
Separated^h^	−.094*** (0.014)	.024* (0.011)	−.010 (0.008)	−.125*** (0.014)	.003 (0.011)	−.001 (0.007)
Children 1–2^i^	−.114*** (0.011)	.001 (0.012)	−.011 (0.007)	−.083*** (0.012)	.010 (0.012)	−.012^+^ (0.007)
Children 3+^i^	−.196*** (0.027)	.015 (0.033)	−.007 (0.020)	−.134*** (0.027)	.003 (0.033)	−.009 (0.019)
*Random effects*						
Var_Residual_	.61			.54		
Var_Ind_	.26			.30		
ICC	.30			.36		
*R* ^2^_Marginal_	.123			.144		
*R* ^2^_Conditional_	.382			.449		

*Note*: *N*_Obs_ = 130,598, *N*_Ind_ = 60,156. Mixed effect regression models with random intercepts for individuals. Standardised estimates and standard errors of main and interaction effects of identity and group variables. Models control for total net income at between‐ and within‐level (logarithmised). Factors with base in brackets: ^a^Gender [male]. ^b^Ethnicity [white]. ^c^Education [basic]. ^d^Occupation [basic]. ^e^Occ. status [employed]. ^f^Party affl. [none]. ^g^Age [<25]. ^h^Civil status [single]. ^i^Children [none].

Abbreviation: ID, identity.

^+^ *p* < .1, *p < .05, **p < .01, ***p < .001.

In contrast, there was clear evidence for the importance of identity domains. The association of educational and political identity with capital income was not only overall positive but also positive – or at least null, never negative – within different educational and political groups, both between and within persons. The association of gender and ethnic identity with capital income was not only overall negative but also negative – or at least null, never positive – within gender and ethnic groups – though only between persons with no associations within persons. Occupational and family identity also showed a consistent association with capital income across occupational and family groups – yet as already mentioned, negative between persons and positive within persons. Only age identity had no consistent association with capital income across age groups, being positive for younger and negative for older individuals (only between persons, no within‐persons associations).

Table 4 summarises the evidence on adherence to norms and the importance of identity domains. It shows that the direction and strength of associations between identity and capital income could align with participation disparities between groups, but this is limited to a few cases related to ethnic, occupational and family identities. In contrast, there was relatively strong evidence for the importance of identity domains. The associations were largely consistent across groups – sometimes weaker but not reversing direction. Educational and political identity had a positive association with capital income within both higher‐ and lower‐capital‐income groups, and especially for those with lower capital income, while gender and ethnic identity had a negative association with capital income within both higher‐ and lower‐capital‐income groups, and especially for those with higher capital income. This suggests a contrasting effect of a socioeconomic and a sociocultural identity domain.

**TABLE 4:** Evidence of norm and domain effects in the link between identity and capital income.

	Overall effect	Norm effect^a^	Domain effect^b^	Pattern description
*Between effects*				
ID gender	Neg.	–	Strong	Neg. for men; no eff. for women
ID ethnicity	Neg.	Weak	Strong	More neg. for Asian, other than White, no eff. for Black
ID education	Pos.	–	Strong	More pos. for lower education levels
ID occupation	Neg./–	Weak	Strong	(More) neg. for higher occ. levels; less neg. for self‐employed, retired than employed
ID politics	Pos.	–	Strong	Pos. for no party affl.; less pos. for Cons., Lib.
ID age	Neg./–	–	–	Pos. for young, neg. for old
ID family	Neg.	–	Strong	Neg. for all civil status/children
*Within effects*				
ID gender	–	–	–	–
ID ethnicity	–	–	–	–
ID education	Pos.	–	Strong	Pos. for all education levels
ID occupation	Pos./–	Weak	Weak	Pos. for skilled, prof., no eff. other occ. levels/status
ID politics	Pos.	–	Weak	Pos. for no party affl., no eff. for other party affl.
ID age	–	–	–	–
ID family	Pos.	Weak	Weak	Pos. for partnered, no eff. for other civil status/children

*Note*: Findings based on Model 1 and Model 2.

Abbreviations: Affl., affiliation; Cons., Conservatives; Eff., effect; ID, identity; Lib., Liberals; Neg., negative; Occ., occupation; Pos., positive; Prof., professionals.

^a^ Norm effect = stronger eff. (weak version) or opposite eff. (strong version) in line with group disparities in capital market participation.

^b^ Domain effect = overall effect (weak version) or same effect across groups (strong version).

### Consistency across income levels

Table 5 illustrates whether the association of social identity with capital income varies by income level (RQ4), based on the regression model including the interaction between social identity and total net income (Model 3). Only singular interaction effects were observed. Between persons, the positive association of political identity with capital income and the negative association of ethnic identity with capital income were stronger at higher income levels (the former only for the amount of capital income). Additionally, the negative association of age identity with capital income was stronger at higher income levels, rendering the main association insignificant. The negative association of family identity with capital income was weaker at higher income levels (only for the likelihood of capital income). No interaction was found at the within‐person level. Overall, identity effects were relatively stable across income levels; all but one association strengthened rather than weakened with increasing income.

**TABLE 5:** Capital income predicted by identities and total income (Model 3).

Predictor	Capital income (any)		Capital income (amount)	
	Main	Int. with income	Main	Int. with income
	*β* (SE)	*β* (SE)	*β* (SE)	*β* (SE)
*Between effects*				
Total income^a^	.084*** (0.004)		.115*** (0.004)	
ID gender	−.016*** (0.004)	.002 (0.004)	−.016*** (0.004)	−.006 (0.004)
ID ethnicity	−.013** (0.004)	−.007* (0.003)	−.020*** (0.004)	−.018*** (0.003)
ID education	.029*** (0.004)	−.003 (0.004)	.030*** (0.004)	−.004 (0.004)
ID occupation	−.015*** (0.004)	.005 (0.003)	−.006 (0.004)	.002 (0.003)
ID politics	.014*** (0.004)	.002 (0.003)	.016*** (0.004)	.010*** (0.003)
ID age	.006 (0.004)	−.011** (0.004)	.001 (0.004)	−.008* (0.004)
ID family	−.038*** (0.003)	.005* (0.003)	−.032*** (0.003)	.003 (0.003)
*Within effects*				
Total income^a^	.032*** (0.002)		.046*** (0.002)	
ID gender	−.002 (0.003)	−.003 (0.003)	−.002 (0.002)	.001 (0.003)
ID ethnicity	−.004 (0.002)	−.002 (0.003)	.000 (0.002)	−.002 (0.003)
ID education	.007** (0.002)	.003 (0.003)	.009*** (0.002)	.004 (0.003)
ID occupation	.006* (0.002)	.000 (0.003)	.001 (0.002)	.000 (0.003)
ID politics	.006* (0.002)	−.001 (0.003)	.005* (0.002)	−.001 (0.003)
ID age	−.001 (0.003)	.006 (0.003)	−.002 (0.002)	.002 (0.003)
ID family	.007** (0.002)	.003 (0.003)	.005* (0.002)	.003 (0.003)
*Random effects*				
Var_Residual_	.61		.54	
Var_Ind_	.26		.30	
ICC	.30		.36	
*R* ^2^_Marginal_	.122		.144	
*R* ^2^_Conditional_	.382		.449	

*Note*: *N*_Obs_ = 130,598, *N*_Ind_ = 60,156. Mixed‐effect regression models with random intercepts for individuals. Main effects and interaction effects (int.) with total income. Standardised estimates and standard errors. Models control for group memberships: gender, ethnicity, education, occupation level and status, party affiliation, civil status and children.

Abbreviation: ID, identity.

^a^ Total monthly individual net income at between and within level (logarithmised).

*p < .05, **p < .01, ***p < .001.

## DISCUSSION

We integrated the social identity approach (Tajfel & Turner, 1979; Turner et al., 1987) with identity economics (Akerlof & Kranton, 2000, 2010), linking psychological measures of social identity to large‐scale, representative UK household data on capital income (60,156 individuals, 130,598 observations, 2010–2023) to examine the association of gender, ethnic, educational, occupational, political, age and family identities with capital income, assessing both societal relevance and variation across income and sociodemographic groups.

In summary, our findings show that social identities – beyond mere group membership – are linked to both the presence and amount of capital income (RQ1). We examined two pathways: adherence to group norms and the importance of identity domains. Evidence for group norms (RQ2) was limited, though in some cases stronger identification in less‐participating groups was associated with lower capital income (e.g., Asian vs. White, employed vs. retirees, partnered vs. not‐partnered). More consistent support emerged for the identity domain pathway (RQ3): socioeconomic identities (i.e., education and politics) were positively associated with capital income, while sociocultural identities (i.e., gender and ethnicity) were negatively associated, both overall and within groups. For example, educational identity was associated with higher capital income across education levels, whereas gender identity was associated with lower capital income especially for men. Finally, the associations between social identity and capital income were stable or even stronger at higher income levels (RQ4). These findings suggest the relevance of identity – in a psychological sense – for capital income across both less‐participating and higher‐income groups.

### Research implications

Our research makes three contributions. First, to identity economics and capital market participation research, it distinguishes social identity, in the psychological sense, from mere group membership. Second, to social identity research, it emphasises the economic consequences of identity and proposes identity domains as a pathway shaping economic behaviour. Third, in the psychology of inequality, it highlights a financial behaviour closely linked to economic inequality but often overlooked in this emerging field.

We show for the first time that social identities are associated with household capital income, beyond typically studied sociodemographic variables. Adding to a psychological perspective (Balloch et al., 2015; Barberis & Thaler, 2003; Donkers & Van Soest, 1999; Salamanca et al., 2020; Van der Heijden et al., 2012) to the economic puzzle of low capital market participation (Haliassos, 2003), we situate financial decision‐making within social context (Hirshleifer, 2015). Extending prior economic research on persisting group disparities in capital income (Barber & Odean, 2001; Bucher‐Koenen et al., 2021; Guiso et al., 2003; Jianakoplos & Bernasek, 1998; Painter et al., 2016), we distinguish social identity from mere group membership (Tajfel & Turner, 1979), also further advancing identity economics (Akerlof & Kranton, 2000, 2010; Charness & Chen, 2020; Shayo, 2020). Unlike studies focusing only on investor identity (Henkel & Zimpelmann, 2023), we examined a broad range of social identities. Using a large representative household dataset, our findings estimate the societal relevance of social identities for real‐life capital income, complementing experimental evidence of their causal role in investment behaviour (Benjamin et al., 2010; Morris et al., 2008). Although their found explanatory value might appear modest, it is comparable to other typically investigated sociodemographic factors.

We further contribute by extending the integration of a social identity approach with identity economics (Steffens et al., 2020), addressing the underexplored economic consequences of social identities (Brown, 2020). Moving beyond traditional group‐norm explanations (Terry et al., 1999), we find evidence for the importance of identity domains as a novel perspective (Easterbrook et al., 2020; McConnell, 2011). Unlike prior research on gender and ethnic disparities in capital income (Jianakoplos & Bernasek, 1998; Painter et al., 2016), we found that gender and ethnic identities were linked with lower capital income, even within groups that typically participate more in capital markets ‐ such as men and White individuals ‐ and that these links persisted or even strengthed at higher income levels. In line with prior research on political engagement (Bonaparte & Kumar, 2013; Changwony et al., 2015), political identity – regardless of party affiliation – was linked to higher capital income, as was educational identity across education levels. Both social identity theory (Tajfel et al., 1971; Tajfel & Turner, 1979) and identity economics (Akerlof & Kranton, 2000, 2010) suggest that social identities can lead individuals to sacrifice economic gains for identity‐related benefits. Research on life satisfaction further supports the idea that sociocultural and socioeconomic identities offer distinct benefits, with communal versus economic identity salience shaping the perceived value of social versus financial resources (Gleibs et al., 2013). Our findings suggest that this dynamic also influences real‐life economic behaviour: individuals with stronger communal or economic identities make different choices regarding capital market participation.

We also uncovered more nuanced associations between social identity and capital income that extend beyond the sociocultural versus socioeconomic distinction. Consistent with the distinction, family identity was associated with lower capital income between individuals, whereas occupational identity was linked to higher capital income within individuals. However, these associations reversed at the opposite level: family identity showed a positive association with capital income within individuals, and occupational identity was negatively associated between individuals. The mechanisms underlying these patterns warrant further research. Stronger family identity may also encourage focus on financial responsibilities and long‐term provision, supporting capital accumulation, while occupational identity may also emphasise labour‐related income and career advancement at the expense of other sources of income such as savings and investments. The latter is particularly relevant to research on occupational identity, which highlights its generally positive effects on job performance, personal well‐being and career outcomes (Greco et al., 2022; Murnieks et al., 2014). Our findings suggest that, in addition to certain community and health costs documented in the literature (Greco et al., 2022), occupational identity may also entail negative economic consequences.

Finally, our research contributes to the emerging field of the psychology of inequality (Jetten & Peters, 2019; Kraus & Stephens, 2012; Manstead, 2018). While research often focuses on the consequences of inequality (Connor et al., 2020; Peters & Jetten, 2023), we add to research examining how psychological factors shape economic behaviour, sustaining inequality (Haushofer & Fehr, 2014; Sheehy‐Skeffington, 2020). We highlight capital market participation, a financial behaviour linked to economic inequality (Piketty, 2014), often overlooked as it occurs among under‐studied higher‐income groups (Leckelt et al., 2019) and reflects privileges rather than disadvantages (Bruckmüller et al., 2017; Phillips et al., 2022). While previous work shows that socioeconomic background structures the self‐concept (Easterbrook et al., 2020) and subjective socioeconomic status influences behaviour (Sheehy‐Skeffington, 2020), we suggest that different identity domains can motivate or constrain economic behaviour across status groups. Socioeconomic identities can encourage economically advantageous actions even in lower‐status groups, whereas sociocultural identities can constrain such behaviour even in higher‐status groups. The persistence of these patterns across income levels suggests an element of choice: while lower‐income groups might face more identity constraints, higher‐income individuals have access to both identity domains. Although our study focuses on the UK, the results might have broader implications for household financial behaviour across Europe and worldwide (EFAMA, 2024; Grout et al., 2009).

### Limitations and future directions

First, while our study found associations between social identity and capital income both between and within persons, causality was not established – though already demonstrated in prior studies (Benjamin et al., 2010; Morris et al., 2008). Unlike experimental work, our findings highlight the societal relevance and complexity of this relationship using real‐world capital income data across diverse sociodemographic backgrounds. Because prior research on capital market participation focused on sociodemographic factors (Barber & Odean, 2001; Bucher‐Koenen et al., 2021; Jianakoplos & Bernasek, 1998; Painter et al., 2016), examining between‐person associations was important to establish the relevance of social identities, in a psychological sense, beyond these traditional factors.

Second, unlike other studies on capital market participation (Balloch et al., 2015; Van Rooij et al., 2011), the UKHLS data do not distinguish between capital income from deposit interest and more deliberate investments, such as shares, funds, or bonds. However, by excluding rents and pensions as capital income, higher capital income in our analysis likely primarily reflects high‐return investments. Future research could explore different types of household investment behaviour through the lens of social identity.

Third, the UKHLS measured identity only by the combination of a domain's importance (e.g., gender) and group categorisation (e.g., man/woman), failing to capture more nuanced identities (e.g., non‐binary gender). Furthermore, the importance of an identity reflects only one aspect of identification. Future work could examine additional components, such as self‐group similarity, group homogeneity, as well as satisfaction and solidarity with a group (Leach et al., 2008). For example, studying whether satisfaction with sociocultural identities is associated with lower capital income could support the idea of accepting economic loss for identity gains.

Fourth, the UKHLS did not assess a financial domain in the self‐concept. While other socioeconomic identities (e.g., education, politics) were predictive, financial identity likely strongly influences capital income. This influence can be positive, as positive perceptions of investors increase investments (Henkel & Zimpelmann, 2023), or negative, as a financial‐focused identity can oppose investment behaviour – as seen with the Occupy Wallstreet Movement (Smith et al., 2015). Future research could examine identity‐driven social actions and their impact on personal finance.

### Practical implications

Our study has practical implications for individuals and society, showing that social identity shapes financial behaviour and outcomes. As Akerlof and Kranton (2000) argue, ‘the choice of identity may be the most important “economic” decision people make’ (p. 717). Individuals should recognise how group norms (e.g., ‘investments aren't for us’) or identity domains (e.g., ‘community matters more than money’) influence financial decisions. Importantly, while social background may seem fixed, actively shaping one's identity can help overcome economic challenges linked to social context.

Policymakers increasingly focus on how financial behaviour affects inequality. For example, the EU is developing a Retail Investment Strategy (European Commission, 2023) under the Capital Market Union Action Plan 2020 to empower retail investors and improve products. Our research offers two policy insights: First, consumer education should go beyond financial literacy (Van Rooij et al., 2011), since knowledge alone does not change behaviour (Thaler, 2016). Addressing psychological factors that drive economic inequality is crucial (Sheehy‐Skeffington & Rea, 2017); financial education should help individuals recognise how social background and identity influence decisions, promoting informed choices and encouraging participation in financial activities such as investing.

Second, investment products should go beyond preventing deceptive marketing or ensuring value for money (European Commission, 2023) and actively reach diverse groups, not just high‐income investors. Identity‐focused interventions can promote sustainable behavior change and broader engagement (Mols et al., 2015; Tarrant et al., 2020). Marketing should include women, ethnic minorities, and community‐oriented groups, signalling that investing is inclusive. Rising interest in sustainable and socially responsible investments provides an opportunity to appeal to previously underrepresented groups (Bauer & Smeets, 2015), enhancing accessibility and inclusivity.

## CONCLUSION

Our research bridges social identity research and identity economics by examining how diverse social identities link to capital market participation and capital income in UK households. Using a psychological measure of social identity alongside traditional sociodemographic variables, we show that group identification – beyond mere membership – significantly predicts financial behaviours and outcomes, contributing to economic inequality. Rather than adherence to group norms, evidence indicates that sociocultural identity domains (e.g., gender, ethnicity) are associated with lower capital income, while socioeconomic identity domains (e.g., education, politics) are linked to higher capital income. These patterns hold across income levels and are comparable in magnitude to traditional socioeconomic factors. By highlighting identity's role in shaping financial behaviour, the study informs policies aimed at reducing capital income disparities and fostering economic inclusivity. Ultimately, our findings emphasise that for individuals and society, there is a price attached to identity.

## AUTHOR CONTRIBUTIONS

**Robin Bachmann:** Conceptualization; methodology; data curation; formal analysis; writing – original draft; writing – review and editing. **Ilka H. Gleibs:** Conceptualization; writing – review and editing; supervision. **Liam Delaney:** Conceptualization; supervision.

## CONFLICT OF INTEREST STATEMENT

The second author serves as Deputy Chief Editor of the British Journal of Social Psychology. To ensure the integrity of the peer review process, the author did not partake in any editorial decisions regarding this manuscript. The manuscript was handled independently by another member of the editorial team, with the author having no access to reviewer identities, reviewer comments, or any editorial correspondence related to this submission. The author had no role in the selection of reviewers or in any editorial decisions pertaining to this manuscript. All review processes were conducted in accordance with the journal's standard double‐blind peer review procedures and ethical guidelines. The authors declare that there are no other competing interests.

## Supporting information


Table S1.
Table S2.

## Data Availability

The data that support the findings of this study are available in the UK Data Service at https://ukdataservice.ac.uk/, reference number 10.5255/UKDA‐SN‐6614‐20. These data were derived from the following resources available in the public domain: University of Essex, Institute for Social and Economic Research (2024). This project was preregistered on its Open Science Framework page (https://osf.io/p43cb).

## Footnotes

^1^
Highest reported education level based on the *International Standard Classification of Education* (*ISCED‐2011*).

^2^
Based on the *Standard Occupational Classification* (*SOC‐2010*), we created four levels – 1: elementary, 2: operative, sales, services, administrative, 3: skilled, associates, 4: professionals, managers.

^3^
‘Which political party did you vote for (in the last general election)?’ Since this question was not included in *Wave 14* (2022–23), we imputed values from the most recent available *Wave 12* (2020–21).

^4^
Number of children under 15 the respondent is responsible for.

^5^
To examine whether between‐ and within‐person identity variations influence both capital income and its growth over time, time was included as a numeric variable with a random slope for each individual (Model 4.1) and subsequently interacted with the identity components (Model 4.2). Results are reported in the supporting information.

^6^
We also examined whether social identities influence capital income growth over time, using a regression model with identity variables interacting with time as a random slope for each individual (Model 4.2; see Table S1). Between‐person effects on growth mirrored the overall associations: ethnic identity was linked to lower capital income and slower growth, whereas educational identity was linked to higher capital income and faster growth (the latter only for likelihood). Within‐person effects showed compensatory dynamics: negative associations at a given point predicted faster growth, whereas positive associations predicted slower growth.

## References

[bjso70025-bib-0001] Akerlof, G. A. , & Kranton, R. E. (2000). Economics and identity. Quarterly Journal of Economics, 115(3), 715–753. 10.1162/003355300554881

[bjso70025-bib-0002] Akerlof, G. A. , & Kranton, R. E. (2010). Identity economics. Princeton University Press.

[bjso70025-bib-0003] Ashforth, B. E. , & Mael, F. (1989). Social identity theory and the organization. Academy of Management Review, 14(1), 20–39. 10.5465/amr.1989.4278999

[bjso70025-bib-0004] Balloch, A. , Nicolae, A. , & Philip, D. (2015). Stock market literacy, trust, and participation. Review of Finance, 19(5), 1925–1963. 10.1093/rof/rfu040

[bjso70025-bib-0005] Barber, B. M. , & Odean, T. (2001). Boys will be boys: Gender, overconfidence, and common stock investment. The Quarterly Journal of Economics, 116(1), 261–292. 10.1162/003355301556400

[bjso70025-bib-0006] Barberis, N. , & Thaler, R. (2003). A survey of behavioral finance. In G. Constantinides , R. M. Stulz , & M. Harris (Eds.), Handbook of the economics of finance: Financial markets and asset pricing (pp. 1053–1128). Elsevier Science & Technology.

[bjso70025-bib-0007] Bauer, R. , & Smeets, P. (2015). Social identification and investment decisions. Journal of Economic Behavior and Organization, 117, 121–134. 10.1016/j.jebo.2015.06.006

[bjso70025-bib-0008] Benjamin, D. J. , Choi, J. J. , & Strickland, A. J. (2010). Social identity and preferences. American Economic Review, 100(4), 1913–1928. 10.1257/aer.100.4.1913 20871741 PMC2944260

[bjso70025-bib-0009] Bonaparte, Y. , & Kumar, A. (2013). Political activism, information costs, and stock market participation. Journal of Financial Economics, 107(3), 760–786. 10.1016/j.jfineco.2012.09.012

[bjso70025-bib-0010] Brown, R. (2020). The social identity approach: Appraising the Tajfellian legacy. British Journal of Social Psychology, 59(1), 5–25. 10.1111/bjso.12349 31691319

[bjso70025-bib-0011] Bruckmüller, S. , Reese, G. , & Martiny, S. E. (2017). Is higher inequality less legitimate? Depends on how you frame it! British Journal of Social Psychology, 56(4), 766–781. 10.1111/bjso.12202 28547801

[bjso70025-bib-0012] Bucher‐Koenen, T. , Alessie, R. J. , Lusardi, A. , & Van Rooij, M. (2021). *Fearless woman: Financial literacy and stock market participation* (ZEW Discussion Paper No. 21‐015). Centre for European Economic Research. 10.2139/ssrn.3798304

[bjso70025-bib-0013] Bucher‐Koenen, T. , & Lusardi, A. (2011). Financial literacy and retirement planning in Germany. Journal of Pension Economics and Finance, 10(4), 565–584. 10.1017/S1474747211000485 PMC5445931 28553190

[bjso70025-bib-0014] Camerer, C. (1999). Behavioral economics: Reunifying psychology and economics. Proceedings of the National Academy of Sciences, 96(19), 10575–10577. 10.1073/pnas.96.19.10575 PMC33745 10485865

[bjso70025-bib-0015] Changwony, F. K. , Campbell, K. , & Tabner, I. T. (2015). Social engagement and stock market participation. Review of Finance, 19(1), 317–366. 10.1093/rof/rft059

[bjso70025-bib-0016] Charness, G. , & Chen, Y. (2020). Social identity, group behavior, and teams. Annual Review of Economics, 12, 691–713. 10.1146/annurev-economics-091619-032800

[bjso70025-bib-0017] Chen, Y. , & Li, S. X. (2009). Group identity and social preferences. American Economic Review, 99(1), 431–457. 10.1257/aer.99.1.431

[bjso70025-bib-0018] Cialdini, R. B. , Reno, R. R. , & Kallgren, C. A. (1990). A focus theory of normative conduct: Recycling the concept of norms to reduce littering in public places. Journal of Personality and Social Psychology, 58(6), 1015–1026. 10.1037/0022-3514.58.6.1015

[bjso70025-bib-0019] Connor, P. , Stancato, D. , Yildirim, U. , Lee, S. , & Chen, S. (2020). Inequality in the minimal group paradigm: How relative wealth and its justification influence ingroup bias. Journal of Experimental Social Psychology, 88, 103967. 10.1016/j.jesp.2020.103967

[bjso70025-bib-0020] Donkers, B. , & Van Soest, A. (1999). Subjective measures of household preferences and financial decisions. Journal of Economic Psychology, 20(6), 613–642. 10.1016/S0167-4870(99)00027-6

[bjso70025-bib-0021] Easterbrook, M. J. , Kuppens, T. , & Manstead, A. S. (2020). Socioeconomic status and the structure of the self‐concept. British Journal of Social Psychology, 59(1), 66–86. 10.1111/bjso.12334 31175690

[bjso70025-bib-0022] European Commission . (2023). Retail investment strategy. European Commission.

[bjso70025-bib-0023] European Fund and Asset Management Association . (2024). Household participation in capital markets. Assessing progress focusing on 2020–2022 .

[bjso70025-bib-0024] Fisman, R. , Paravisini, D. , & Vig, V. (2017). Cultural proximity and loan outcomes. American Economic Review, 107(2), 457–492. 10.1257/aer.20120942

[bjso70025-bib-0025] Gleibs, I. H. , Morton, T. A. , Rabinovich, A. , Haslam, S. A. , & Helliwell, J. F. (2013). Unpacking the hedonic paradox: A dynamic analysis of the relationships between financial capital, social capital and life satisfaction. British Journal of Social Psychology, 52(1), 25–43. 10.1111/j.2044-8309.2011.02035.x 21623839

[bjso70025-bib-0026] Gomila, R. (2021). Logistic or linear? Estimating causal effects of experimental treatments on binary outcomes using regression analysis. Journal of Experimental Psychology: General, 150(4), 700–709. 10.1037/xge0000920 32969684

[bjso70025-bib-0027] Greco, L. M. , Porck, J. P. , Walter, S. L. , Scrimpshire, A. J. , & Zabinski, A. M. (2022). A meta‐analytic review of identification at work: Relative contribution of team, organizational, and professional identification. Journal of Applied Psychology, 107(5), 795–830. 10.1037/apl0000941 34591563

[bjso70025-bib-0028] Grout, P. A. , Megginson, W. L. , & Zalewska, A. A. (2009). One half‐billion shareholders and counting‐determinants of individual share ownership around the world . SSRN. 10.2139/ssrn.1364765

[bjso70025-bib-0029] Guiso, L. , Haliassos, M. , & Jappelli, T. (2003). Stockholding: A European comparison. In L. Guiso , M. Haliassos , & T. Jappelli (Eds.), Stockholding in Europe (pp. 3–29). Springer.

[bjso70025-bib-0030] Guo, P. , Shi, G. , Tian, G. G. , & Duan, S. (2021). Politicians' hometown favoritism and corporate investments: The role of social identity. Journal of Banking and Finance, 125, 106092. 10.1016/j.jbankfin.2021.106092

[bjso70025-bib-0031] Haliassos, M. (2003). Stockholding: Recent lessons from theory and computations. In L. Guiso , M. Haliassos , & T. Jappelli (Eds.), Stockholding in Europe (pp. 30–49). Springer.

[bjso70025-bib-0032] Haslam, C. , Jetten, J. , Cruwys, T. , Dingle, G. , & Haslam, S. A. (2018). The new psychology of health: Unlocking the social cure (1st ed.). Routledge.

[bjso70025-bib-0033] Haslam, S. A. (2001). Psychology in organizations: The social identity approach. Sage Publications. 10.4135/9781446278819

[bjso70025-bib-0034] Haushofer, J. , & Fehr, E. (2014). On the psychology of poverty. Science, 344(6186), 862–867. 10.1126/science.1232491 24855262

[bjso70025-bib-0035] Henkel, L. , & Zimpelmann, C. (2023). *Proud to not own stocks: How identity shapes financial decisions* (IZA Discussion Paper No. 206). Institute of Labor Economics. 10.2139/ssrn.4490155

[bjso70025-bib-0075] Hirshleifer, D. (2015). Behavioral finance. Annual Review of Financial Economics, 7(1), 133–159. 10.1146/annurev-financial-092214-043752

[bjso70025-bib-0036] Jetten, J. , & Peters, K. (2019). The social psychology of inequality. Springer.

[bjso70025-bib-0037] Jianakoplos, N. A. , & Bernasek, A. (1998). Are women more risk averse? Economic Inquiry, 36(4), 620–630. 10.1111/j.1465-7295.1998.tb01740.x

[bjso70025-bib-0038] Kahneman, D. , & Tversky, A. (1979). Prospect theory: An analysis of decision under risk. Econometrica, 47(2), 263–291. 10.2307/1914185

[bjso70025-bib-0039] Kraus, M. W. , & Stephens, N. M. (2012). A road map for an emerging psychology of social class. Social and Personality Psychology Compass, 6(9), 642–656. 10.1111/j.1751-9004.2012.00453.x

[bjso70025-bib-0040] La Rue, C. J. , Steffens, N. K. , Werth, B. Á. , Bentley, S. V. , & Haslam, C. (2024). A latent profile analysis of the nature of social group memberships and their contribution to retirement outcomes. British Journal of Social Psychology, 63(2), 591–613. 10.1111/bjso.12694 37905751

[bjso70025-bib-0041] Leach, C. W. , van Zomeren, M. , Zebel, S. , Vliek, M. L. , Pennekamp, S. F. , Doosje, B. , Ouwerkerk, J. W. , & Spears, R. (2008). Group‐level self‐definition and self‐investment: A hierarchical (multicomponent) model of in‐group identification. Journal of Personality and Social Psychology, 95(1), 144–165. 10.1073/pnas.1811987116 18605857

[bjso70025-bib-0042] Leckelt, M. , Richter, D. , Schröder, C. , Küfner, A. C. , Grabka, M. M. , & Back, M. D. (2019). The rich are different: Unravelling the perceived and self‐reported personality profiles of high‐net‐worth individuals. British Journal of Psychology, 110(4), 769–789. 10.1111/bjop.12360 30466138

[bjso70025-bib-0043] Manstead, A. S. (2018). The psychology of social class: How socioeconomic status impacts thought, feelings, and behaviour. British Journal of Social Psychology, 57(2), 267–291. 10.1111/bjso.12251 29492984 PMC5901394

[bjso70025-bib-0044] McConnell, A. R. (2011). The multiple self‐aspects framework: Self‐concept representation and its implications. Personality and Social Psychology Review, 15(1), 3–27. 10.1177/1088868310371101 20539023

[bjso70025-bib-0045] McNeish, D. , & Kelley, K. (2019). Fixed effects models versus mixed effects models for clustered data: Reviewing the approaches, disentangling the differences, and making recommendations. Psychological Methods, 24(1), 20–35. 10.1037/met0000182 29863377

[bjso70025-bib-0046] Mols, F. , Haslam, S. A. , Jetten, J. , & Steffens, N. K. (2015). Why a nudge is not enough: A social identity critique of governance by stealth. European Journal of Political Research, 54(1), 81–98. 10.1111/1475-6765.12073

[bjso70025-bib-0047] Mols, F. , Haslam, S. A. , Platow, M. J. , Reicher, S. D. , & Steffens, N. K. (2023). The social identity approach to political leadership. In L. Huddy , D. O. Sears , J. S. Levy , & J. Jerit (Eds.), The Oxford handbook of political psychology (3rd ed., pp. 804–842). Oxford University Press.

[bjso70025-bib-0048] Morris, M. W. , Carranza, E. , & Fox, C. R. (2008). Mistaken identity: Activating conservative political identities induces ‘conservative’ financial decisions. Psychological Science, 19(11), 1154–1160. 10.1111/j.1467-9280.2008.02217.x 19076488

[bjso70025-bib-0049] Murnieks, C. Y. , Mosakowski, E. , & Cardon, M. S. (2014). Pathways of passion: Identity centrality, passion, and behavior among entrepreneurs. Journal of Management, 40(6), 1583–1606. 10.1177/0149206311433855

[bjso70025-bib-0050] Painter, M. A. , Holmes, M. D. , & Bateman, J. (2016). Skin tone, race/ethnicity, and wealth inequality among new immigrants. Social Forces, 94(3), 1153–1185. 10.1093/sf/sov094

[bjso70025-bib-0051] Peters, K. , & Jetten, J. (2023). How living in economically unequal societies shapes our minds and our social lives. British Journal of Psychology, 114(2), 515–531. 10.1111/bjop.12632 36708128

[bjso70025-bib-0052] Phillips, L. T. , Jun, S. , & Shakeri, A. (2022). Barriers and boosts: Using inequity frames theory to expand understanding of mechanisms of race and gender inequity. Academy of Management Annals, 16(2), 547–587. 10.5465/annals.2020.0314

[bjso70025-bib-0053] Piketty, T. (2014). Capital in the twenty‐first century. Harvard University Press. 10.1111/1468-4446.12115 25516350

[bjso70025-bib-0054] Reicher, S. , & Hopkins, N. (2000). Self and nation. Sage.

[bjso70025-bib-0055] Salamanca, N. , de Grip, A. , Fouarge, D. , & Montizaan, R. (2020). Locus of control and investment in risky assets. Journal of Economic Behavior and Organization, 177, 548–568. 10.1016/j.jebo.2020.06.032

[bjso70025-bib-0056] Shayo, M. (2020). Social identity and economic policy. Annual Review of Economics, 12, 355–389. 10.1146/annurev-economics-082019-110313

[bjso70025-bib-0057] Sheehy‐Skeffington, J. (2020). The effects of low socioeconomic status on decision‐making processes. Current Opinion in Psychology, 33, 183–188. 10.1016/j.copsyc.2019.07.043 31494518

[bjso70025-bib-0058] Sheehy‐Skeffington, J. , & Rea, J. (2017). How poverty affects people's decision‐making processes. Joseph Rowntree Foundation.

[bjso70025-bib-0059] Smith, L. G. , Gavin, J. , & Sharp, E. (2015). Social identity formation during the emergence of the occupy movement. European Journal of Social Psychology, 45(7), 818–832. 10.1002/ejsp.2150

[bjso70025-bib-0060] Steffens, N. K. , Haslam, S. A. , Peters, K. , & Quiggin, J. (2020). Identity economics meets identity leadership: Exploring the consequences of elevated CEO pay. The Leadership Quarterly, 31(3), 101269. 10.1016/j.leaqua.2018.10.001

[bjso70025-bib-0061] Struewing, C. , & Jirjahn, U. (2019). *Does society influence the gender gap in risk attitudes? Evidence from east and west Germany* (IZA Discussion Paper No. 12100). Institute of Labor Economics 10.2139/ssrn.3390088

[bjso70025-bib-0062] Tajfel, H. (1981). Human groups and social categories: Studies in social psychology. Cambridge University Press.

[bjso70025-bib-0063] Tajfel, H. , Billig, M. G. , Bundy, R. P. , & Flament, C. (1971). Social categorization and intergroup behaviour. European Journal of Social Psychology, 1(2), 149–178. 10.1002/ejsp.2420010202

[bjso70025-bib-0064] Tajfel, H. , & Turner, J. C. (1979). An integrative theory of intergroup conflict. In W. G. Austin & S. Worchel (Eds.), The social psychology of intergroup relations (pp. 33–37). Brooks/Cole.

[bjso70025-bib-0065] Tarrant, M. , Haslam, C. , Carter, M. , Calitri, R. , & Haslam, S. A. (2020). Social identity interventions. In K. Hamilton , L. D. Cameron , M. S. Hagger , N. Hankonen , & T. Lintunen (Eds.), The handbook of behavior change (pp. 649–660). Cambridge University Press.

[bjso70025-bib-0066] Terry, D. J. , Hogg, M. A. , & White, K. M. (1999). The theory of planned behaviour: Self‐identity, social identity and group norms. British Journal of Social Psychology, 38(3), 225–244. 10.1348/014466699164149 10520477

[bjso70025-bib-0067] Thaler, R. H. (2016). Behavioral economics: Past, present, and future. American Economic Review, 106(7), 1577–1600. 10.1257/aer.106.7.1577

[bjso70025-bib-0068] Turner, J. C. , Hogg, M. A. , Oakes, P. J. , Reicher, S. D. , & Wetherell, M. S. (1987). Rediscovering the social group: A self‐categorization theory. Basil Blackwell.

[bjso70025-bib-0069] UK Department for Work and Pensions . (2022). *Family resources survey 2020/21. Savings and investments* [Data set].

[bjso70025-bib-0070] UK Office of National Statistics . (2023). *The effects of taxes and benefits on household income, disposable income estimate. Financial year ending 2022* [Data set].

[bjso70025-bib-0071] University of Essex, Institute for Social and Economic Research . (2024). *Understanding society: Waves 1–14, 2009–2023* (19th ed.) [Data set]. UK Data Service. 10.5255/UKDA-SN-6614-20

[bjso70025-bib-0072] Van der Heijden, E. , Klein, T. J. , Müller, W. , & Potters, J. (2012). Framing effects and impatience: Evidence from a large scale experiment. Journal of Economic Behavior and Organization, 84(2), 701–711. 10.1016/j.jebo.2012.09.017

[bjso70025-bib-0073] Van Rooij, M. , Lusardi, A. , & Alessie, R. (2011). Financial literacy and stock market participation. Journal of Financial Economics, 101(2), 449–472. 10.1016/j.jfineco.2011.03.006

[bjso70025-bib-0074] Walasek, L. , & Brown, G. D. (2015). Income inequality and status seeking: Searching for positional goods in unequal US states. Psychological Science, 26(4), 527–533. 10.1177/0956797614567511 25792131

