# Supplementary material for: Social identity and capital income: A social psychological approach to identity economics using UK household data
Source: Br J Soc Psychol. 2025 Dec 3;65(1):e70025. doi: 10.1111/bjso.70025 (PMC12673441; doi:10.1111/bjso.70025)
Supplement: Supplementary file 1 — Table S1. Table S2. [file BJSO-65-0-s001.docx]

**Tables**

| **Table S1**  *Capital income predicted by identities with time effects (Model 4)* | | | | | | | |
| --- | --- | --- | --- | --- | --- | --- | --- |
|  | Capital income (any) | | | Capital income (amount) | | |  |
|  | Model 4.1 | Model 4.2 | | Model 4.1 | Model 4.2 | |  |
|  | Main | Main | Int. with time | Main | Main | Int. with time |  |
| Predictor | *β (SE)* | *β (SE)* | *β (SE)* | *β (SE)* | *β (SE)* | *β (SE)* |  |
| Time | -.018*** (.002) | .016 (.010) |  | -.007*** (.002) | .019+ (.010) |  |  |
| *Between effects* | | | | | | |  |
| ID gender | -.017*** (.004) | -.013 (.008) | -.001 (.003) | -.016*** (.004) | -.015+ (.008) | .000 (.002) |  |
| ID ethnicity | -.013** (.004) | .001 (.008) | -.006* (.002) | -.018*** (.004) | -.003 (.008) | -.006* (.002) |  |
| ID education | .027*** (.004) | .015+ (.008) | .005* (.002) | .030*** (.004) | .022** (.008) | .003 (.002) |  |
| ID occupation | -.015*** (.004) | -.015* (.007) | .000 (.002) | -.006 (.004) | -.004 (.007) | .000 (.002) |  |
| ID politics | .016*** (.004) | .016* (.007) | -.001 (.002) | .016*** (.004) | .009 (.007) | .002 (.002) |  |
| ID age | .007+ (.004) | .000 (.008) | .003 (.002) | .001 (.004) | -.009 (.008) | .004 (.002) |  |
| ID family | -.040*** (.003) | -.041*** (.007) | .001 (.002) | -.032*** (.003) | -.033*** (.006) | .001 (.002) |  |
| *Within effects* | | | | | | |  |
| ID gender | -.003 (.003) | -.007 (.007) | .002 (.002) | -.002 (.002) | -.003 (.006) | .001 (.002) |  |
| ID ethnicity | -.004+ (.002) | -.011+ (.006) | .002 (.002) | -.001 (.002) | -.007 (.006) | .002 (.002) |  |
| ID education | .005+ (.002) | -.018** (.006) | .008*** (.002) | .007** (.002) | -.010+ (.006) | .006** (.002) |  |
| ID occupation | .005+ (.002) | .007 (.006) | -.001 (.002) | .001 (.002) | .002 (.006) | .000 (.002) |  |
| ID politics | .010*** (.002) | .025*** (.006) | -.005** (.002) | .006** (.002) | .020*** (.006) | -.005* (.002) |  |
| ID age | .000 (.003) | .012 (.006) | -.004 (.002) | -.002 (.002) | .006 (.006) | -.003 (.002) |  |
| ID family | .005* (.002) | -.014* (.006) | .006** (.002) | .004* (.002) | -.005 (.006) | .003+ (.002) |  |
| *Random effects* | | | | | | |  |
| *Var_Residual_* | .59 | .59 |  | .51 | .51 |  |  |
| *Var_Ind_* | .33 | .32 |  | .38 | .37 |  |  |
| *Var_Time_* | .01 | .01 |  | .01 | .01 |  |  |
| *Corr* | -.46 | -.45 |  | -.46 | -.45 |  |  |
| *ICC* | .32 | .32 |  | .39 | .39 |  |  |
| *R^2^_Marginal_* | .122 | .124 |  | .143 | .144 |  |  |
| *R^2^_Conditional_* | .401 | .401 |  | .480 | .479 |  |  |
| *Note. N_Obs_* = 130,598, *N_Ind_* = 60,156. Mixed effect regression models with random intercepts and time slopes for individuals. Main effects and interaction effects (int.) with time. Standardized estimates and standard errors. Models control for total net income at between- and within-level (logarithmized) as well as group memberships: gender, ethnicity, education, occupation level and status, party affiliation, civil status, and children. *ID* = identity. +*p* < .1. **p* < .05. ***p* < .01. ****p* < .001. | | | | | | | |

| **Table S2**  *Capital income predicted by identities within groups (Model 2)* | | | | |
| --- | --- | --- | --- | --- |
|  | Capital income (any) | | Capital income (amount) | |
|  | ID_between_ | ID_within_ | ID_between_ | ID_within_ |
| Group | *β (SE)* | *β (SE)* | *β (SE)* | *β (SE)* |
| *ID gender* |  |  |  |  |
| Male^a^ | -.028*** (.005) | -.006+ (.003) | -.029*** (.005) | -.005 (.003) |
| Female^a^ | -.008 (.005) | .000 (.003) | -.005 (.005) | .000 (.003) |
| *ID ethnicity* |  |  |  |  |
| White^b^ | -.007 (.005) | -.004 (.003) | -.015*** (.005) | -.001 (.002) |
| Black^b^ | -.023 (.016) | .006 (.012) | -.017 (.016) | -.003 (.011) |
| Asian^b^ | -.043*** (.012) | -.007 (.008) | -.031** (.012) | .004 (.008) |
| Other^b^ | -.064* (.029) | -.032 (.022) | -.099*** (.029) | -.032 (.021) |
| *ID education* |  |  |  |  |
| Basic^c^ | .043*** (.006) | .010** (.004) | .045*** (.006) | .008* (.004) |
| Alevel^c^ | .021*** (.006) | .008* (.004) | .017** (.006) | .010** (.004) |
| Uni^c^ | .019** (.007) | .003 (.004) | .024*** (.007) | .007+ (.004) |
| *ID occupation* |  |  |  |  |
| Elementary^d^ | -.011 (.013) | .010 (.010) | -.004 (.013) | -.007 (.009) |
| Administrative^d^ | -.050*** (.008) | .005 (.005) | -.036*** (.008) | -.003 (.005) |
| Skilled^d^ | -.029** (.010) | .011+ (.007) | -.028** (.010) | .010 (.006) |
| Professionals^d^ | -.020* (.010) | .015** (.006) | -.020* (.010) | .006 (.005) |
| Employed^e^ | -.011 (.013) | .010 (.010) | -.004 (.013) | -.007 (.009) |
| Self-employed^e^ | .011 (.017) | -.002 (.012) | .022 (.017) | -.013 (.011) |
| Unemployed^e^ | .020 (.020) | -.011 (.016) | .005 (.020) | -.020 (.015) |
| Not-working^e^ | .032* (.015) | .008 (.013) | .027+ (.015) | -.003 (.012) |
| Retired^e^ | .022 (.016) | .009 (.012) | .024 (.016) | .000 (.011) |
| *ID politics* |  |  |  |  |
| None^f^ | .016*** (.004) | .009** (.003) | .015*** (.004) | .006* (.003) |
| Conservative^f^ | -.003 (.010) | -.001 (.005) | .024* (.010) | .008 (.005) |
| Labour^f^ | .024** (.008) | .006 (.005) | .018* (.008) | .001 (.005) |
| Liberal^f^ | -.007 (.016) | .005 (.009) | -.016 (.017) | -.002 (.009) |
| Green^f^ | -.012 (.025) | -.005 (.015) | .016 (.025) | .014 (.014) |
| Other^f^ | -.004 (.019) | -.016 (.018) | .003 (.019) | -.010 (.017) |
| *ID age* |  |  |  |  |
| <25^g^ | .029** (.009) | -.006 (.010) | .032*** (.009) | -.009 (.010) |
| 25-39^g^ | .030*** (.007) | .003 (.005) | .026*** (.007) | -.007 (.005) |
| 40-54^g^ | .006 (.007) | -.003 (.004) | .003 (.007) | .000 (.004) |
| 55-70^g^ | -.011 (.007) | .000 (.004) | -.019** (.007) | -.004 (.004) |
| 70<^g^ | -.024** (.009) | .005 (.006) | -.040*** (.009) | .007 (.006) |
| *ID family* |  |  |  |  |
| Single^h^ | -.042*** (.006) | .005 (.005) | -.027*** (.006) | .007 (.005) |
| Couple^h^ | -.043*** (.008) | .012+ (.006) | -.030*** (.008) | .009 (.006) |
| Partnered^h^ | -.036*** (.005) | .011*** (.003) | -.035*** (.005) | .007* (.003) |
| Separated^h^ | -.018+ (.009) | -.005 (.006) | -.024* (.009) | .005 (.006) |
| No children^i^ | -.042*** (.006) | .005 (.005) | -.027*** (.006) | .007 (.005) |
| 1-2 children^i^ | -.040** (.013) | -.005 (.008) | -.017 (.013) | -.005 (.008) |
| 3+ children^i^ | -.027 (.033) | -.002 (.021) | -.024 (.033) | -.003 (.019) |
| *Note. N_Obs_* = 130,632, *N_Ind_* = 60,186. Standardized estimates and standard errors of identity effects within groups based on mixed effect regression models with interactions between identity and group variables (Model 2). Shown also in Figure 1. Models control for total net income at between- and within-level (logarithmized). Groups: ^a^gender. ^b^ethnicity. ^c^education. ^d^occupation. ^e^occ. status. ^f^party affl.. ^g^age. ^h^civil status. ^i^children. +*p* < .1. **p* < .05. ***p* < .01. ****p* < .001. | | | | |
